# Clinical validation of a multiplex droplet digital PCR for diagnosing suspected bloodstream infections in ICU practice: a promising diagnostic tool

**DOI:** 10.1186/s13054-022-04116-8

**Published:** 2022-08-08

**Authors:** Jing Wu, Bin Tang, Yuzhen Qiu, Ruoming Tan, Jialin Liu, Jiang Xia, Jing Zhang, Jingjing Huang, Jieming Qu, Jingyong Sun, Xiaoli Wang, Hongping Qu

**Affiliations:** 1 Department of Critical Care Medicine, Ruijin Hospital, Shanghai Jiao Tong University School of Medicine, No. 197 Ruijin Er Road, Shanghai, 200025 China; 2 Pilot Gene Technologies (Hangzhou) Co., Ltd, No. 688 Bin’an Road, Hangzhou, 310051 China; 3 Department of Pharmacy, Ruijin Hospital, Shanghai Jiao Tong University School of Medicine, Shanghai, 200025 China; 4 Department of Pulmonary and Critical Care Medicine, Ruijin Hospital, Shanghai Jiao Tong University School of Medicine, No.197 Ruijin Er Road, Shanghai, 200025 China; 5 Department of Laboratory Medicine, Ruijin Hospital, Shanghai Jiao Tong University School of Medicine, No. 197 Ruijin Er Road, Shanghai, 200025 China

**Keywords:** Clinical validation, Droplet digital PCR, Bloodstream infections (BSIs), Critically ill patients, Discordant results

## Abstract

**Background:**

Droplet digital PCR (ddPCR) has emerged as a promising tool of pathogen detection in bloodstream infections (BSIs) in critical care medicine. However, different ddPCR platforms have variable sensitivity and specificity for diverse microorganisms at various infection sites. There is still a lack of prospective clinical studies aimed at validating and interpreting the discrepant ddPCR results for diagnosing BSI in intensive care unit (ICU) practice.

**Methods:**

A prospective diagnostic study of multiplex ddPCR panels was conducted in a general ICU from May 21, 2021, to December 22, 2021. Paired blood cultures (BCs) and ddPCRs (2.5 h) were obtained synchronously to detect the 12 most common BSI pathogens and three antimicrobial resistance (AMR) genes. Firstly, ddPCR performance was compared to definite BSI. Secondly, clinical validation of ddPCR was compared to composite clinical diagnosis. Sensitivity, specificity, and positive and negative predictive values were calculated. Thirdly, the positive rate of AMR genes and related analysis was presented.

**Results:**

A total of 438 episodes of suspected BSIs occurring in 150 critical patients were enrolled. BC and ddPCR were positive for targeted bacteria in 40 (9.1%) and 180 (41.1%) cases, respectively. There were 280 concordant and 158 discordant. In comparison with BCs, the sensitivity of ddPCR ranged from 58.8 to 86.7% with an aggregate of 72.5% in different species, with corresponding specificity ranging from 73.5 to 92.2% with an aggregate of 63.1%. Furthermore, the rate of ddPCR+/BC− results was 33.6% (147/438) with 87.1% (128 of 147) cases was associated with probable (*n* = 108) or possible (*n* = 20) BSIs. When clinically diagnosed BSI was used as true positive, the final sensitivity and specificity of ddPCR increased to 84.9% and 92.5%, respectively. In addition, 40 *bla*_KPC_, 3*bla*_NDM_, and 38 *mec*A genes were detected, among which 90.5% were definitely positive for *bla*_KPC_. Further, 65.8% specimens were predicted to be *mec*A-positive in *Staphylococcus sp.* according to all microbiological analysis.

**Conclusions:**

The multiplexed ddPCR is a flexible and universal platform, which can be used as an add-on complementary to conventional BC. When combined with clinical infection evidence, ddPCR shows potential advantages for rapidly diagnosing suspected BSIs and AMR genes in ICU practice.

**Supplementary Information:**

The online version contains supplementary material available at 10.1186/s13054-022-04116-8.

## Background

Bacterial bloodstream infections (BSIs) and associated sepsis/septic shock are one of the leading causes of mortality in critically ill patients whose physical and immune barriers are disrupted [1]. This condition is further complicated by the increasing global burden of antimicrobial resistance and inappropriate use of antibiotic drugs [2]. International Guidelines for Management of Sepsis and Septic Shock 2021 recommend the administration of antimicrobials immediately, ideally within 1 h of diagnosis [3]. Therefore, rapid diagnosis and early administration of appropriate antimicrobials are crucial determinants in improving prognosis and decreasing the all-cause mortality rate in BSIs, especially in the case of drug-resistant bacterial infections [1]. Conventional blood culture (BC) is the gold standard for causative pathogen identification and antimicrobial susceptibilities test (AST) in the diagnosis of BSIs. However, this method is limited by suboptimal sensitivity ranging from ≤ 10% to about 50% in patients with suspected bacteremia, febrile neutropenia, or sepsis/septic shock [4]. This is primarily due to low levels of circulating microorganisms, slow growing microorganisms, and long turnaround times. Hence, the development of accurate BSI diagnostic tools for rapid pathogen detection and antibiotic stewardship in endemic regions such as emergency departments and intensive care units (ICUs) is a top priority.

In contrast to molecular tests performed on bacterial isolates, attempts have been made to directly detect pathogens and resistance markers in blood samples without prior incubation to improve BSI diagnosis [5, 6], including multiplex real-time polymerase chain reaction (PCR)-based MagicPlex® Sepsis Test [7], PCR combined with T2 magnetic resonance-based T2Candida [8] and T2Bacteria panel [9], metagenomics-based assays (LightCycler®SeptiFast) [10], and even next-generation sequencing [11]. However, several of these technologies are still limited and have been abandoned in the clinical environment because of medium sensitivity/specificity, high operational costs, and incapable of performing ASTs [6, 12]. Recently, digital polymerase chain reactions (dPCRs), the third-generation PCR after real-time quantitative PCR (qPCR), have been developed to offer a number of technical advantages to address these challenges [13, 14]. Mechanically speaking, a PCR Master Mix was divided into thousands of partitions using emulsified microdroplets suspended in oil (i.e., droplet digital PCR; ddPCR), followed by PCR amplification of target genes in each individual partition, thereby acting as an individual microreactor. In summary, ddPCR is less affected by PCR inhibitors and more sensitive to microbial genes, thus leading to higher sensitivity and precision. Moreover, this technology offers high reproducibility and provides absolute quantification without the need for a standard curve [15]. Various dPCR platforms have been developed in different areas [16–18]. In the field of critical care medicine, the functions of dPCR are attractive and have been proposed as a potential tool for pathogen identification in blood or other clinical samples, severity assessment, prognosis, treatment guidance, and profile host responses to infection [15]. Nevertheless, there is still a lack of prospective studies aimed to validate ddPCR performance in BSI diagnosis in ICU clinical practice.

In this study, we used a multiplex ddPCR panel to detect the most clinically relevant pathogens and related resistance genes in critically ill patients with suspected BSIs with a turnaround time of 2.5 h. We identified eight bacterial species involved with the most common “ESKAPE” pathogens (*Enterococcus faecium*, *Staphylococcus aureus*, *Klebsiella pneumoniae*, *Pseudomonas aeruginosa*, and *Escherichia coli*), as well as four fungi species (*Candida parapsilosis*, *Candida tropicalis*, *Candida glabrata*, and *Candida albicans*) and three resistance markers (*bla*_KPC_*, bla*_NDM_, and *mecA*). Herein, a single-center prospective study was performed in an integrated ICU to evaluate the concordance between ddPCR and conventional BC results. It is worth noting that the explanation of discrepancies results between the ddPCR and BC remains controversial. Notably, the disaccord results and clinical utility of ddPCR for diagnosing suspected BSIs were comprehensively interpreted according to all microbiological cultures and clinical evidence [9].

## Methods

### Study population

**Fig. 1:**
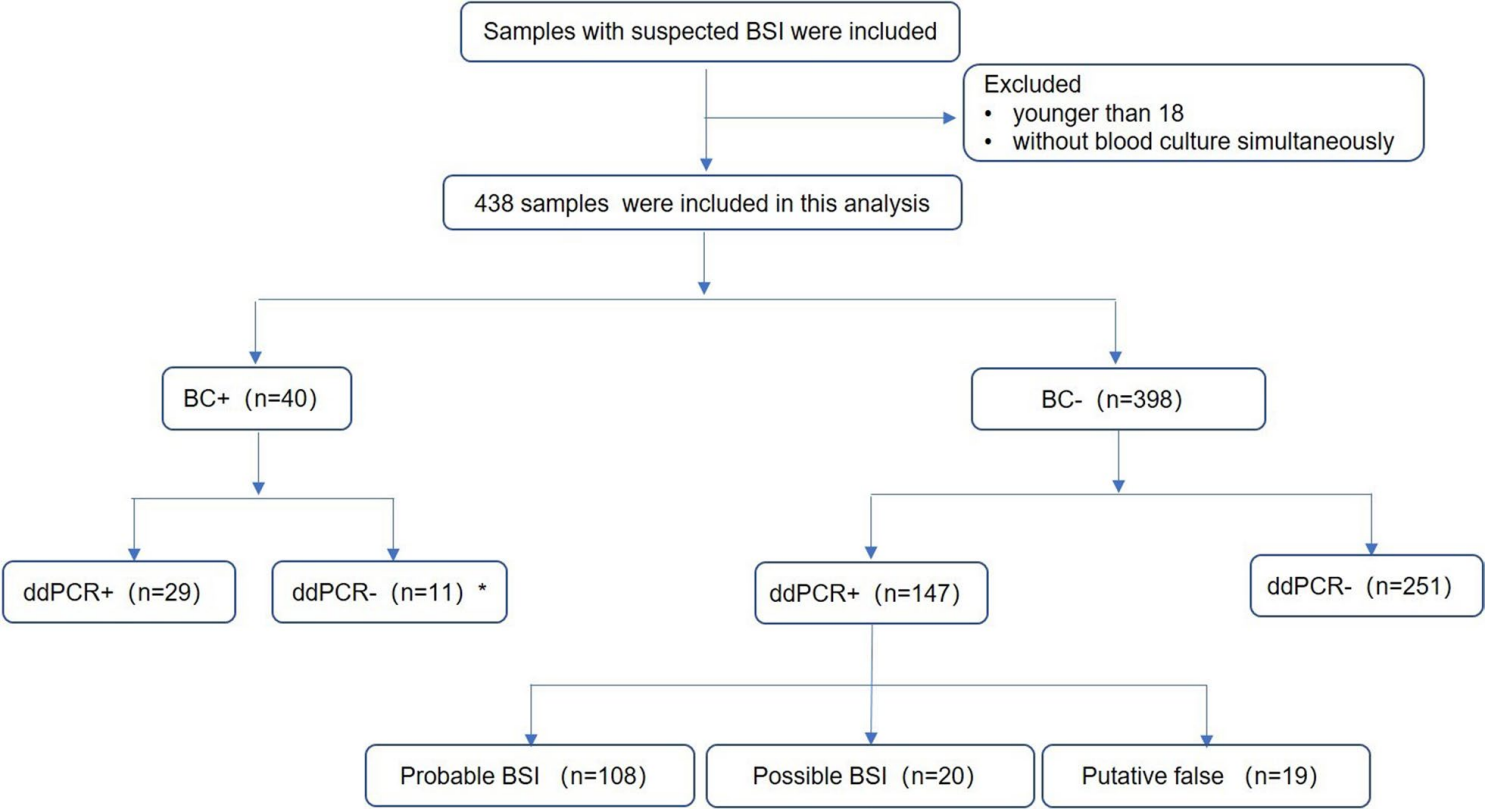
Flow chart for patient enrollment and results analysis. *In four samples, the blood culture and ddPCR testing showed different targeted bacteria concurrently; these ddPCR cases were defined as presumptive false-negative

This study was a prospective pilot diagnostic study to clinically validate the multiplex ddPCR panel in diagnosing suspected BSIs in critically ill patients. This work was performed in the integrated ICU of Ruijin Hospital, Shanghai Jiao Tong University School of Medicine, from May 21, 2021, to December 22, 2021. Critically ill patients (older than 18 years) with suspected BSIs were eligible and consecutively recruited for the study (Fig. 1). Patients with mental disorders and pregnant women were excluded from the study. Contaminated or damaged samples were also eliminated. The study protocol allowed the inclusion of multiple episodes of suspected BSI occurring in one patient.

### Sample collection and BSI diagnosis

Upon clinical suspicion of a BSI by training ICU physicians, two sets of blood cultures (both aerobic and anaerobic bottles, 10–20 mL per bottle) and at least 3 mL whole blood samples (EDTA blood collection tubes) were obtained from the same catheter or venipuncture for BSI diagnosis and ddPCR testing. Blood cultures were incubated for a maximum of 5 days (BD BACTEC FX; BD Biosciences), and pathogens in positive cultures were identified using matrix-assisted laser desorption ionization-time of flight mass spectrometry (bioMérieux, Marcy-l’Étoile, France) [19]. ASTs were performed using the VITEK2 compact system and interpreted according to the Clinical and Laboratory Standards Institute guidelines (M100-ED30) [20]. Conventional culturing results were analyzed in terms of pathogen detection and resistance patterns. Strains that showed resistance to imipenem or meropenem were identified as carbapenem-resistant. PCRs were performed to detect carbapenem-encoding resistance genes (*bla*_KPC_ and *bla*_NDM_) [21]. Methicillin-resistant *Staphylococcus sp*. was speculated *mec*A-positive.

### Plasma DNA Extraction and ddPCR testing

**Fig. 2:**
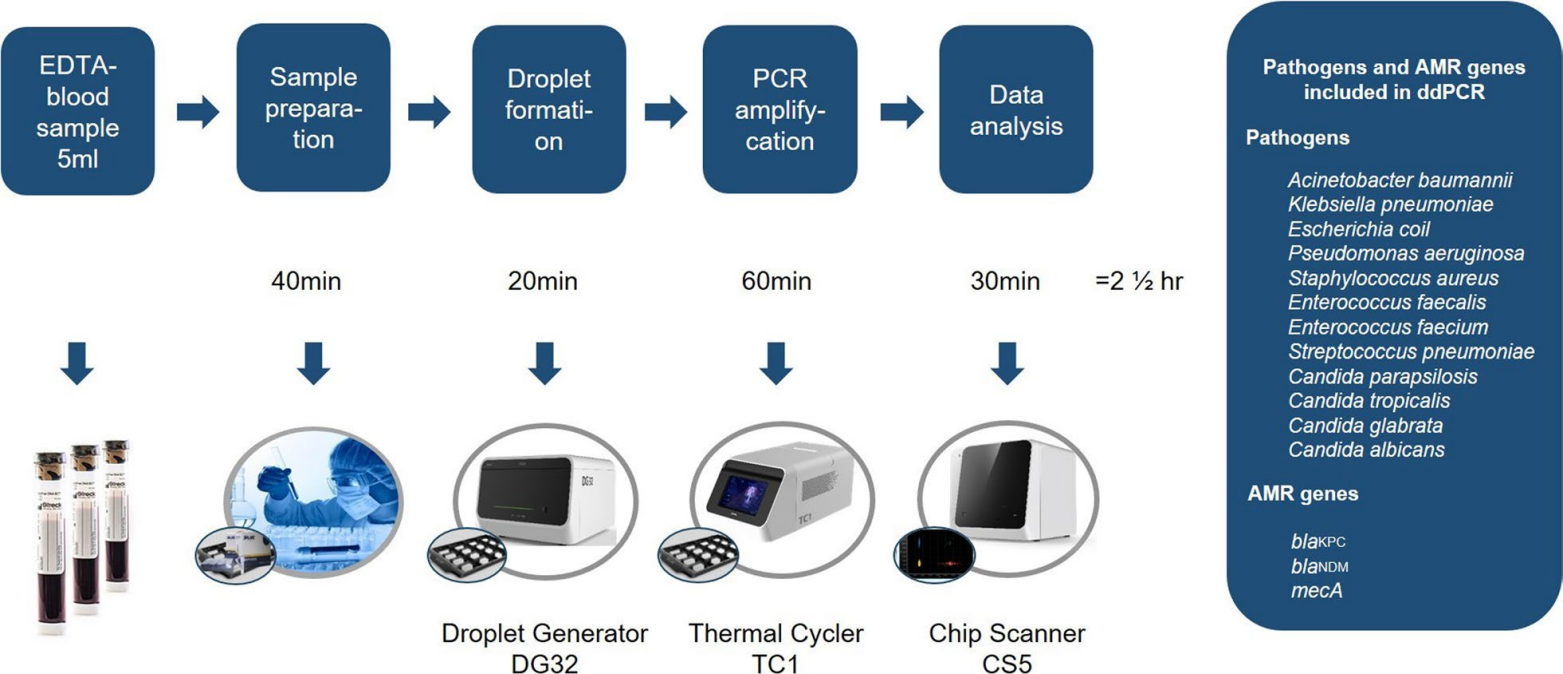
Droplet digital PCR detection process, pathogens, and AMR genes detected were included. EDTA, ethylenediamine tetraacetic acid; AMR genes, antimicrobial resistance genes

The multiplex ddPCR testing, which consists of five channels, allows for the detection of the eight most common bacterial pathogens, four fungal pathogens, and three antimicrobial resistance (AMR) genes directly from blood in amounts as small as 50 copies/mL (Pilot Gene Technologies. Hangzhou, China) (Fig. 2) [22]. The detection limit was determined by ddPCR detection of whole blood specimens spiked with multiple known concentrations of different microbial species. We found the detection sensitivity of ddPCR to be 50 copy units per mL (copies/mL), with the exception of *bla*_KPC_ (80 copies/mL). Whole-blood samples were stored at 4 °C, and ddPCR testing was conducted on the same day or on the next day because of the timing of suspected BSI. Further, ddPCR testing procedures were performed for about 2.5 h according to the manufacturer’s protocol (Fig. 2). Samples were processed to plasma by centrifugation (1,600 r.c.f. for 15 min), and sample preparation took about 40 min. Next, the reaction mixture in the sample cup was passed through the micro-channel (Droplet Generator DG32) under the action of pressure, and tens of thousands of water-in-oil emulsion droplets were generated due to gravity and shear force in 20 min. After PCR amplification for 60 min by Thermal Cycler TC1, scanning and data analysis for droplet counts and amplitudes were performed within 30 min using a chip scanner CS5 and GenePMS software (v2.0.01.20011). The synthesized DNA fragment was used as positive control, and DNase-free water or blood samples from three healthy subjects were used as negative controls. The copies of each targeted pathogen or gene were reported by ddPCR results.

### Definitions and clinical data

Culture-proven BSI is defined by positive blood cultures in a patient with systemic signs of infection and may be either secondary to a documented source or primary, according to the definitions released by National healthcare safety network (https://www.cdc.gov/nhsn/pdfs/pscmanual/4psc_clabscurrent) [1]. Clinical data were extracted from the patients’ medical records, including the demographic, comorbidities, organ dysfunction, surgical intervention, and clinical outcomes. Severity of disease was assessed by the Sequential Organ Failure Assessment (SOFA) and Acute Physiology and Chronic Health Evaluation II (APACHE II) scoring system. Results of ddPCRs and BCs were independently verified by two ICU physicians. Suspected infectious episodes of all routine microbiological cultures, including BC, abdominal, respiratory, skin and soft tissue, and other tissue/fluid cultures, were collected within 7 days of enrolment according to the standard microbiology laboratory procedures. A composite clinical infection standard was defined, consisting of all microbiological results plus clinical adjudication [9, 23].

### Interpretation of BSI and ddPCR results

BSI results involved in the ddPCR-targeted pathogens or AMR genes were summarized for further data analysis. The ddPCR result was considered positive if 1 or more target bacteria were detected and negative if none were detected. Polymicrobial infection was defined as an episode in which more than one microorganism was detected by either ddPCR or blood culture. The BSI and ddPCR results were classified as concordant (both positive and negative) or discordant. The cases in which BCs were positive but ddPCR results were negative or different were defined as presumptive false-negative cases. To resolve discrepancies, discordant ddPCR+/BSI− results were classified as probable BSI, possible BSI, or presumptive false-positive cases similar to previous studies [9, 23]. The following definitions were used for each classification, (i) probable: ddPCR result was concordant with a microbiological test performed within seven days of sample collection from other extra-blood site; (ii) possible: without microbiological data but ddPCR result had potential for pathogenicity based on clinical presentation and laboratory findings; (iii) presumptive false-positive: ddPCR result was inconsistent with clinical presentation.

### Statistical analysis

Primary outcomes were the sensitivity and specificity of ddPCR testing, which were calculated by comparing positive BC results with the ddPCR-targeted pathogens and AMR genes. The secondary outcomes were the clinical validation of the ddPCR testing for diagnosing suspected BSIs, which were compared with all microbiological cultures and the composite clinical diagnosis. Sensitivity, specificity, and positive and negative predictive values were calculated using those findings. For per-assay calculations, results for individual pathogens in each sample were considered separately. Statistical analysis was performed with IBM SPSS Statistics software (v 23.0) (IBM, Armonk, NY, USA). Continuous variables were expressed as the median and interquartile range (IQR). Categorical variables were reported as frequencies and percentages. The difference in positivity rate between BCs and ddPCRs was explored with the Chi-square test. Differences were considered to be statistically significant for if *P* values were ≤ 0.05.

## Results

### Patient clinical characteristics and BC results

**Fig. 3:**
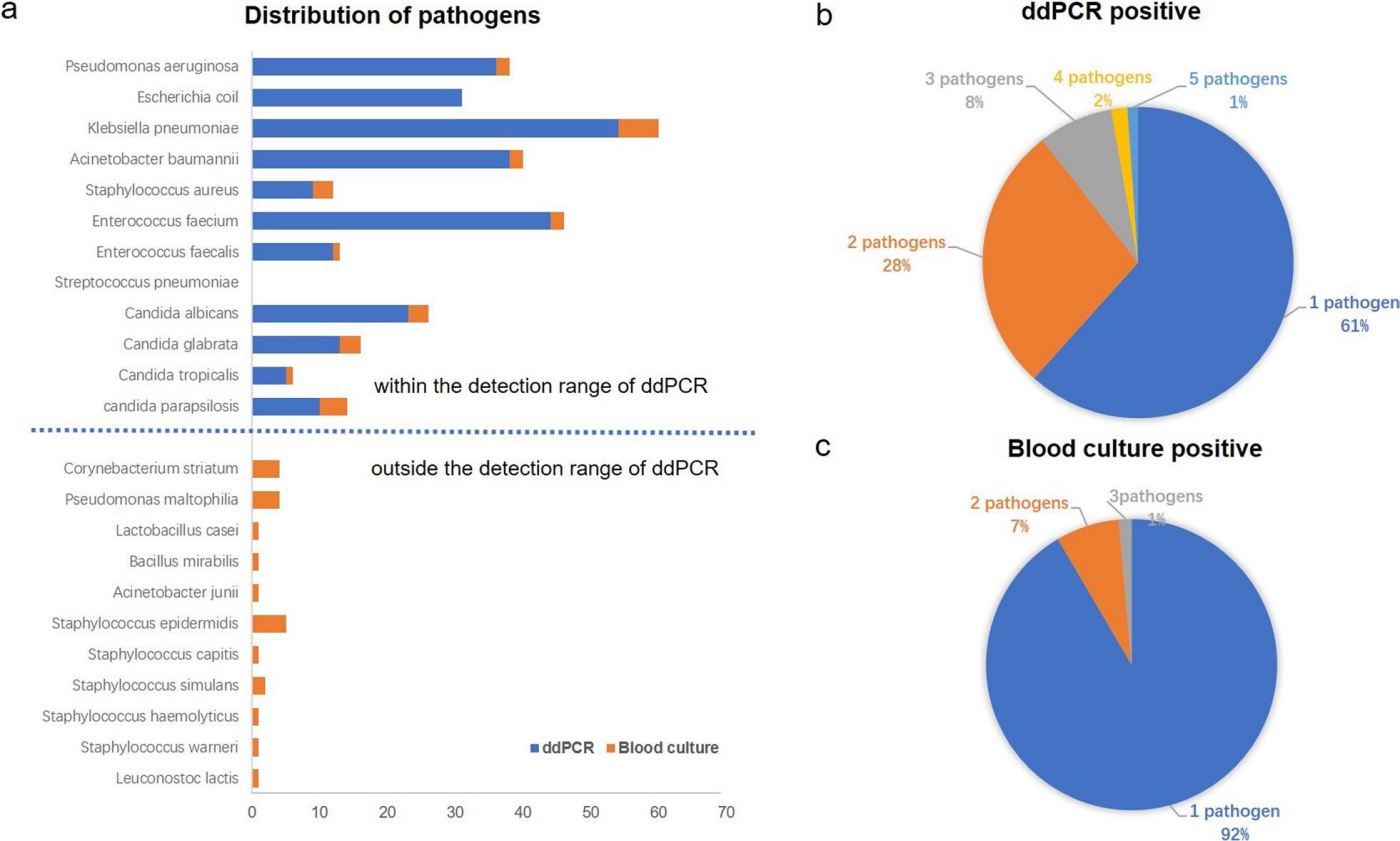
Distribution of pathogens detected by blood culture and ddPCR testing. **a** Pathogens detected by ddPCR and blood culture within and outside the range of ddPCR targeted organisms. Counts and percentages of co-infections in patients of **b** ddPCR-positive and **c** blood culture-positive results

In total, 438 episodes (BC and ddPCR simultaneously) of suspected BSIs occurring in 150 critically ill patients were consecutively recruited, of which 27 patients contributed two episodes and 13 patients three episodes of suspected BSI, and even more episodes involved in several patients (Table 1 and Fig. 1). Table 1 reports the underlying diseases and outcomes of the 150 patients. Among them, 40.7% were thought to be suffering septic shock and treated with vasopressors. The cumulative mortality rate at 28 day was 16.7%. Culture-proven BSI was positive for 78 microorganisms in 16.2% (71 of 438) episodes; polymicrobial BSI was detected in 8.0% (6 of 71) of cases (Fig. 3c). Pathogens included in the ddPCR panel were identified in 56.3% (40 of 71) of positive BCs, 37.5% positives for Gram-negative bacteria, 20.0% for Gram-positive bacteria, and 42.5% for fungi, including *Acinetobacter baumannii* (*n* = 2), *K. pneumonia* (*n* = 9), *E. coil* (*n* = 1), *P. aeruginosa* (*n* = 3), *S. aureus* (*n* = 3), *Enterococcus faecalis* (*n* = 1), *E. faecium* (*n* = 4), *C. parapsilosis* (*n* = 5), *C. tropicalis* (*n* = 1), *C. glabrata* (*n* = 5), and *C. albicans* (*n* = 6). Further, coagulase-negative *staphylococci*, *Pseudomonas maltophilia*, *Acinetobacter junii* and other species, were recovered from the remaining 43.7% BCs (Fig. 3a).

**Table 1:** Clinical characteristics of the critically ill patients

Clinical characteristics	*N* = 150
Age, years, [median (IQR)]	66 (58, 76)
Male, *n* (%)	99 (66.0)
Comorbidities	
Hypertension, *n* (%)	85 (56.7)
Diabetes mellitus, *n* (%)	48 (32.0)
Coronary heart disease, *n* (%)	25 (16.7)
CKD, *n* (%)	26 (17.3)
Malignant tumor, *n* (%)	60 (40.0)
COPD, *n* (%)	4 (2.7)
Immunosuppressive, *n* (%)	22 (14.7)
Mechanical ventilation, *n* (%)	88 (58.7)
Renal replacement therapy, *n* (%)	19 (12.7)
Treated with vasopressors, *n* (%)	61 (40.7)
Surgery performed before 14 days of inclusion, *n* (%)	100 (67.7)
SOFA score, [median (IQR)]	7 (3, 10)
APACHE II score, [median (IQR)]	19 (12, 25)
28-day mortality, *n* (%)	25 (16.7)

IQR, interquartile range; CKD, chronic kidney disease; COPD, chronic obstructive pulmonary disease; SOFA, Sequential Organ Failure Assessment; APACHE II, Acute Physiology and Chronic Health Evaluation II

### Performance of the ddPCR testing

We found that ddPCR results were positive in 41.1% (180 of 438) of episodes, with identification of 275 pathogens (Table 2). Polymicrobial infections were detected in 38.3% (69 of 180) of episodes of ddPCR testing (Fig. 3b). Among them, 159 Gram-negative bacteria detected, with the top three strains being *K. pneumoniae* (*n* = 54), *A. baumannii* (*n* = 38), and *P. aeruginosa* (*n* = 36). In addition, the 65 Gram-positive pathogens detected by ddPCR included *E. faecium* (*n* = 44), *E. faecalis* (*n* = 12), *S. aureus* (*n* = 9). Further, the most detected fungi in the remaining 51 strains were *C. albicans* (*n* = 23), *C. glabrata* (*n* = 13), *C. tropicalis* (*n* = 5), and *C. parapsilosis* (*n* = 10). The performance of the ddPCR method in diagnosing culture-proven BSIs caused by targeted bacteria is summarized in Fig. 3a and Table 2. In eight and two episodes, a second or third organism was also identified by ddPCR testing, and four organisms in one episode, indicating the difficulty of diagnosing polymicrobial infections in conventional BC method. Compared to BC results, 72.5% (29/40) of positive ddPCR tests were also positive in BC. However, this meant ddPCR failed to detect causative pathogens in 11 episodes, which were defined as presumably false-negative cases, including *K. pneumoniae* (*n* = 1), *P. aeruginosa* (*n* = 1), *E. faecium* (*n* = 1), *S. aureus* (*n* = 1), *C. parapsilosis* (*n* = 4), *C. glabrata* (*n* = 1), and *C. albicans* (*n* = 2). In four samples of the 11 episodes, ddPCR detected *E. coli, K. pneumoniae, C. parapsilosis,* and *C. albicans* pathogens, which were different in BC results as *E. faecium*, *C. parapsilosis, C. albicans*, and *K. pneumoniae* were detected instead, respectively.

**Table 2:** Performance of ddPCR results for targeted organisms

	BC+/ddPCR+, n*	BC+/ddPCR−, n**	BC−/ddPCR+, *n*	BC−/ddPCR−, n***
Pathogens (all)	44	11	231	251
*Acinetobacter baumannii*	3	0	35	–
*Klebsiella pneumoniae*	9	1	45	–
*Escherichia coli*	5	0	26	–
*Pseudomonas aeruginosa*	5	1	31	–
*Staphylococcus aureus*	3	1	6	–
*Enterococcus faecalis*	3	0	9	–
*Enterococcus faecium*	5	1	39	–
*Streptococcus pneumoniae*	0	0	0	–
*Candida parapsilosis*	1	4	9	–
*Candida tropicalis*	1	0	4	–
*Candida glabrata*	5	1	8	–
*Candida albicans*	4	2	19	–

*The results of BC+/ddPCR+, BC+/ddPCR−, and BC−/ddPCR+ were calculated according to pathogens. For the BC+/ddPCR+ group, there were polymicrobial infections in 11 cases by ddPCR testing

**In four samples of the 11 BC-positive episodes, ddPCR testing showed difference with the BC results, which were comprehensively analyzed combined with clinical presentation

***Episode (sample) with the

### Overall concordance between BCs and ddPCRs

Results of BC and ddPCR were concordantly positive in 29 episodes, concordantly negative in 251 episodes, and discordant in 158 episodes (Fig. 1). The level of agreement between culture-proven BSIs and ddPCR was 63.9% (280/438). For culture-proven BSIs, the aggregate ddPCR testing demonstrated a sensitivity of 72.5%, a specificity of 63.1%, a positive predictive value (PPV) of 16.5%, and a negative predictive value (NPV) of 95.8%. However, the sensitivity of individual ddPCR detection channels within the multiplex setting was highly variable at genus or gram stain level. Separately, the sensitivity of ddPCR-targeted Gram-negative bacteria was 86.7%, which was higher than 75.0% for Gram-positive bacteria and 58.8% for fungi. Correspondingly, the specificity of ddPCR testing in comparison with the BC method ranged from 73.5 to 92.2% across the detection channels (Table 3).

**Table 3:** Positive and negative agreement of ddPCR versus BC, all microbiological testing, and clinical diagnosis within the detection range of ddPCR

	Sample (*n* = 438)	ddPCR+	ddPCR−	Sensitivity (%)	Specificity (%)	PPV (%)	NPV (%)
Total	Positive by blood culture	29	11	72.5	63.1	16.5	95.8
	Negative by blood culture	147	251				
G^−^	Positive by blood culture	13	2	86.7	73.5	10.4	99.4
	Negative by blood culture	112	311				
G^+^	Positive by blood culture	6	2	75.0	88.8	11.1	99.5
	Negative by blood culture	48	382				
Fungi	Positive by blood culture	10	7	58.8	92.2	23.3	98.2
	Negative by blood culture	33	388				
Positive by all microbiological testing		137	181	43.1	67.5	77.8	30.9
Negative by all microbiological testing		39	81				
Positive by clinical diagnosis		157	28	84.9	92.5	89.2	89.3
Negative by clinical diagnosis		19	234				

G^−^, Gram-negative bacteria; G^+^, Gram-positive bacteria; PPV, positive predictive value, NPV, negative predictive value

### Evaluation of patients with discordant results between BC and ddPCR

The ddPCR results were positive in 33.6% (147 of 438) of episodes with negative BCs, which embraced 231 targeted organisms (Table 2). When we analyzed these 147 discordant results in-depth, combining composite microbiological and clinical evidence, 108 episodes (73.5%) met criteria for probable BSI, 20 episodes (13.6%) for possible BSI, and the remaining 19 cases (12.9%) were presumptive false positives (Fig. 1). Among the probable/possible BSIs, discordant results were often associated with patients diagnosed with earlier or subsequent BCs and localized infections according to pathogens summarized in Additional file 1: Table S1 [blood culture (*n* = 38), abdominal (*n* = 68), respiratory (*n* = 17), skin and soft tissue (*n* = 3), perianal (*n* = 11), urine (*n* = 2), and multiple sites (*n* = 11)]. The ultimately diagnostic performance of ddPCR testing is presented in Table 3. Meanwhile, the 69 polymicrobial infections detected by ddPCR have been further interpreted combined with the composite microbiological and clinical infection evidence. 37.7% episodes (26/69) were completely concordant with all microbiological testing within seven days. 55.1% episodes (38/69) were partly concordant with all microbiological testing. In addition, in comparison with all microbiological testing, 63.4% (104/164) of the polymicrobial ddPCR+ pathogens were also detected in other infection sites. 20.7% (34/164) fulfilled the criteria for possible BSI, and the remaining 15.9% (26/164) were defined as presumptive false-positive cases. If considering probable BSIs with all microbiological testing, the sensitivity and specificity of ddPCR testing were 43.1% and 67.5%, respectively, and the corresponding PPV and NPV were 77.8% and 30.9%, respectively. If considering both probable and possible BSIs were assumed to be true positive, anticipated sensitivity and specificity, as well as the PPV and NPV of ddPCR for complicated BSIs would increase to 84.9%, 92.4%, 89.2%, and 89.3%, respectively.

In summary, the sensitivity of ddPCR for suspected BSI was higher than that of conventional BCs in the 438 cases (35.8% vs 9.1*%, P* < 0.001). Also, we noticed in the 147 ddPCR+/BC− episodes, 44.9% (66/147) received targeted antimicrobial therapy, 27.9% (41/147) received partial targeted treatment owing to the polymicrobial infections, and 27.2% (40/147) did not receive any appropriate treatment before the BC and ddPCR tests. These preliminary data suggest that ddPCR has potentiality to rapidly identify and exclude targeted pathogens, including cases that may be missed by conventional BCs or inhibited by prior antibiotics applications.

### Evaluation of the AMR genes detected by ddPCR

In our research, ddPCR showed 40 episodes positive for *bla*_KPC_, among which, *K. pneumoniae* and *bla*_KPC_ gene were simultaneously detected in 75.0% cases, which were considered more meaningful in the clinical environment (Table 4). Compared with BC results, six cases of BC-reported *K. pneumoniae* showed resistance to carbapenems, and PCR results showed that these strains expressed the *bla*_KPC_ gene. Considering pathogens recovered from other microbiological testing and AST, all cases were resistant to carbapenems, among which 21 strains of *K. pneumoniae* were stored and detected, with 90.5% (19/21) of samples expressing the *bla*_KPC_ gene. As for the *bla*_NDM_ gene, while positive in three cases, no causative pathogen was detected according to BC or other microbiological testing. Further, 38 episodes were positive of *mec*A genes in ddPCR testing. Only three samples were both positive for *S. aureus* and the *mec*A gene, but this could not be confirmed by BC (Table 4). However, 23.7% cases were predicted as *mec*A-positive in *S. aureus* combined with all microbiological and AST results. In addition, 10 (26.3%) and 25 (65.8%) cases were predicted as *mec*A-positive in other *Staphylococcus sp.* that were not included by the ddPCR panel according to the BC and other microbiological testing, respectively.

**Table 4:** AMR genes detected by ddPCR and the related pathogens detected by blood culture and all microbiological testing

AMR genes	Pathogens	ddPCR+ *n*, (%)	BC+ and according to AST, *n*, (%) *	Microbiological testing and, according to AST *n*, (%)
*bla*_*KPC*_ (*n* = 40)	*klebsiella pneumoniae*	30 (75.0)	6 (15.0)	40 (100)
	None	10 (25.0)	34 (85.0)	0 (0)
*bla*_*NDM*_ (*n* = 3)	None	3 (100)	3(100)	3(100)
*mecA* (*n* = 38)	*Staphylococcus aureus*	3 (7.9)	0	9 (23.7)
	*Staphylococcus haemolyticus*	–	2 (5.3)	5 (13.2)
	*Staphylococcus capitis*	–	2 (5.3)	2 (5.3)
	*Staphylococcus epidermidis*	–	3 (7.9)	6 (15.8)
	*Staphylococcus hominis*	–	1 (2.6)	1 (2.6)
	*Staphylococcus simulans*	–	1 (2.6)	1 (2.6)
	*Staphylococcus warneri*	–	1 (2.6)	1 (2.6)
	None		28 (73.7)	13 (34.2)

*Staphylococcus haemolyticus and *Staphylococcus epidermidis* were detected by blood culture in one sample. AMR gene, antimicrobial resistance gene; CRKP, carbapenem-resistant *Klebsiella pneumoniae*

## Discussion

Clinical management of BSIs in critically ill patients poses several challenges because signs and symptoms are generally nonspecific. Studies showed that about 35.0% of patients with sepsis are culture-negative, and Gupta et al*.* reported that mortality was significantly higher in these BC-negative patients [24]. Our study showed that the mortality rate of ddPCR+/BC− patients was 26.2%, similar to ddPCR+/BC+ patients, significantly higher than the ddPCR-/BC- group in Additional file 1: Table S2. Therefore, it is still necessary to take other appropriate diagnostic methods as add-ons complementary to conventional BC to identify the possible causative pathogens for BC-negative sepsis patients. Although numerous biomarkers have been explored to assist in the rapid diagnosis of serious infections, causative pathogen-related molecular diagnostic strategies that directly detect multiple species and resistance phenotypes in whole blood samples without the need for cultivating organisms are urgently needed to distinguish causative infection and define non-infectious inflammatory states in the context of sepsis. Based on the strengths of the ddPCR method, several studies have explored its application in infectious diseases [13]. In the EPICIII study, 41.41% gram-negative microorganisms, 43.17% gram-positive microorganisms, and 10.56% fungal microorganisms were detected among patients admitted to ICUs with proven BSI [25]. Therefore, we designed the multiplex ddPCR panel according to the global and local pathogen epidemiology.

Clinical validation has revealed that the ddPCR method is a flexible and universal platform, but this was at small-scale and not routinely used in the urgent ICU environment [26–30]. Nevertheless, like other molecular tests such as metagenomics-based assays, ddPCR detects microbial Cell-Free DNA (mcfDNA) in plasma, but unable to distinguish mcfDNA between live microorganisms and apoptotic microorganisms. However, mcfDNA could be continuously detected in patients with bloodstream infection or sepsis and evaluated through dynamic monitoring [31]. Also, the clinical significance of ddPCR testing in rapidly pathogens diagnosis was not clearly clarified in critically ill patients [25]. Further, ddPCR shows potential advantages over BC in terms of sensitivity and specificity when combined with clinical infection evidence among the 150 critically ill patients in our study. There were 280 (63.9%) concordant positive or negative results between the two methods. In addition, ddPCR and BC result showed that 180 (41.1%) and 40 (9.1%) of the 438 cases of BSIs yielded targeted bacterial pathogens, respectively. Among the 40 BC+ cases, 11 pathogens were missed by the ddPCR test, the overall sensitivity of ddPCR in comparison with the BC results ranged from 58.8% in fungi to 86.7% in gram-negative bacteria, with an aggregate sensitivity of 72.5%. The corresponding specificity was 92.2% and 73.5%, with an aggregate specificity of 63.1%. Importantly, discordant ddPCR+/BC− results represented 33.6% (147/438) of all reported tests, and the detailed review of clinical circumstances showed that the majority of discordant results were either probable (24.7%,108/438) or possible (4.6%,20/438) BSIs. When the clinically diagnosed BSIs criterion was used as the comparator, the overall sensitivity and specificity of ddPCR increased to 84.9% and 92.5%, respectively. The final sensitivity of ddPCR for suspected BSI was higher than that of conventional BC (35.8% vs 9.1*%, P* < 0.001). In addition, ddPCR also allowed for the detection of AMR genes including *bla*_KPC_*, bla*_NDM_*,* and *mec*A. We found that 90.5% (19/21) of cases were definitely positive for *bla*_KPC,_ no causative pathogen was identified for *bla*_NDM_, and 65.8% cases are predicted as *mec*A-positive in *Staphylococcus sp.* according to all microbiological, AST, and PCR tests. Nonetheless, the application of ddPCR for AMR genes needs further verification.

The causative pathogens in our multiplex ddPCR panel generally covered about 60.0% of organisms recovered from BCs. Furthermore, ddPCR shows higher rate for polymicrobial infection episodes (38.3% vs 8.0*%, P* < 0.001); this is difficult for BC method, which regularly detects only fastest-growing microorganisms [32]. The observed sensitivity of ddPCR is different for various types of microorganisms; although it is relatively high for the majority of individual Gram-negative bacteria, it is insufficient for yeasts. In addition, six fungi were missing and defined as presumptive false-negative cases. Similar results were found in Wouters’ study for identifying 20 bacteria and six fungi, and the sensitivity of identifying Gram-positive and Gram-negative bacteria by ddPCR was 71.0% and 67.0%, respectively, but lower for fungi (60.0%) [33]. In fact, higher sensitivities for fungal detection have been previously reported by other BSI molecular assays such as the T2Candida [9]. Indeed, during our initial ddPCR development, fungal detection was deemed satisfactory in spiking experiments using laboratory-grown organisms. This was partly because of the differences in the methodology such as lysis process, and the success rate of extracting bacterial nucleic acid from blood samples by the ddPCR system was between 15 and 80% [27]. Fungi derived from clinical blood samples may be more difficult to process due to the changes in cell wall characteristics [34]. It is likely that low circulating DNA concentrations in false-negative samples were below the limit of detection of the assay. However, further evaluation and collaborative studies of flexible ddPCR panels covering different pathogens in species or genus level and related resistance genes should be designed based on epidemiological surveillance data in local ICU wards.

Previous studies showed that 10–40% of negative BCs were found to be positive using multiplex molecular assays, and there is no consensus about the interpretation of BC−/ddPCR+ results [35, 36]. We deliberately focused on the clinical interpretation of discrepant ddPCR-positive results, which, to the best of our knowledge, has not yet been addressed in the literature. Consequently, future informed decision-making will have to consider whether a positive ddPCR result represents a true BSI in the context of additional factors, including clinical, epidemiological, and other laboratory data [9, 11]. The possible reasons of BC−/ddPCR+ result might be explained by the presence of nonviable, nonproliferating, or transient or intermittent bacteremia, intracellular organisms within circulating phagocytic cells, inhibition of bacterial growth by antibiotics, or possible contamination [9]. In our study, 73.5% (108/140) BC−/ddPCR+ cases were thought to be probable BSIs because of transient or intermittent bacteremia. The same pathogen was recovered from previous and sequent blood/non-blood site cultures (mainly abdominal infection). Moreover, 14.3% (20/140) of BC−/ddPCR+ cases were considered as possible BSIs because they fit the clinical infection syndromes. Taken together, the sensitivity and specificity of ddPCR were higher than BCs, meaning that ddPCRs could be used as a tool for early detection of suspected BSIs with extra-blood site infections missed by BCs. Although the sensitivity and specificity of ddPCR were reasonable in our study, a higher sensitivity and specificity are desirable in clinical practice. This situation could be countered by dynamic monitoring or investigative ddPCRs from BCs after several hours of incubation [37, 38].

Recently, metagenomic next-generation sequencing (mNGS) has also shown promising potential as a diagnostic tool for BSIs [39]. Hu et al*.* reported that ddPCR was more rapid and sensitive than mNGS within the detection range of 20 common isolated pathogens and four AMR genes, while mNGS detected a broader range of pathogens than ddPCR [22]. The frequent detection of contaminants and colonizing pathogens both affects the specificity of NGS and ddPCR and complicates the interpretation of results in diagnosing BSIs. However, the clinical applications of these two methods play vital roles in different clinical scenes. Further, ddPCR showed a great potential to identify and exclude the common BSI pathogens, whereas mNGS is more appropriate in the diagnosis of rare infections and intractable diseases. In addition, the availability of a bioinformatics analysis team, a typical turnaround time of 2 days for processing and interpreting the sequencing data, and the high cost of NGS represents barriers to the application of NGS in clinical practice.

In this study, 40 *bla*_KPC,_ 3 *bla*_NDM_, and 38 *mec*A genes were detected, meaning that AMR gene detection is more sensitive than pathogen detection in ddPCR testing. This may be due to untargeted ranges or low bacterial loads. As resistance mechanisms are not a definite proof of AST, ddPCR cannot replace it but represents a promising diagnosis method that is complementary to BC, which has imperfect sensitivity in critically ill patients [40]. Moving forward, ddPCR may also have particular value in conjunction with cultures during antimicrobial therapy and rational antimicrobial management strategies, including severity stratification. The impact of ddPCR-based interventions and optimizing treatment and patients’ outcomes requires further clinical research [15, 41].

Several limitations should be underlined. Firstly, this was a monocentric prospective study, and our results warrant further investigations. Secondly, because of the economic cost, ddPCR was simultaneously collected with only a single set of BC samples and generally identified a limited spectrum of microorganisms and AMR genes. However, the testing cost will be further reduced with the technical development and widespread application of ddPCR for diagnosing suspected BSIs. Thirdly, because several patients were repeatedly enrolled, the antimicrobial treatment impact on BC and ddPCR positivity were not performed. Finally, the correlations between quantitative ddPCR results and severity stratification inferred to pathogen load were not analyzed.

## Conclusions

The multiplex ddPCR can be used as add-on complementary assay to the conventional BC method and offered some added diagnostic value for rapidly and accurately diagnosing common suspected BSIs and related AMR genes in ICU practices. Development of ddPCR assays including flexible pathogens and resistance determinants according to local epidemiology are required before it can be used as a precise bedside test.

## Supplementary Information

**Additional file 1.** **Table S1.** Detailed descriptions of the inconsistency BC−/ddPCR+ cases. **Table S2.** Clinical outcomes of the 150 critically ill patients according to ddPCR and BC results.

## Data Availability

Data supporting the conclusions of this article are included within the article and its additional files. The datasets used and/or analyzed in the current study are available from the corresponding author on reasonable request.

## Abbreviations

ddPCR: Droplet digital PCR
BSIs: Bloodstream infections
ICU: Intensive care unit
BC: Blood culture
AMR: Antimicrobial resistance
ASTs: Antimicrobial susceptibility tests
PCR: Polymerase chain reaction
dPCRs: Digital polymerase chain reactions
qPCR: Quantitative PCR
SOFA: Sequential Organ Failure Assessment
APACHE II: Acute Physiology and Chronic Health Evaluation II
IQR: Interquartile range
PPV: Positive predictive value
NPV: Negative predictive value
mNGS: Metagenomic next-generation sequencing
