# Supplementary material for: Clinical validation of a multiplex droplet digital PCR for diagnosing suspected bloodstream infections in ICU practice: a promising diagnostic tool
Source: Crit Care. 2022 Aug 8;26:243. doi: 10.1186/s13054-022-04116-8 (PMC9358819; doi:10.1186/s13054-022-04116-8)
Supplement: Supplementary file 1 — Additional file 1. Table S1. Detailed descriptions of the inconsistency BC−/ddPCR+ cases. Table S2. Clinical outcomes of the 150 critically ill patients according to ddPCR and BC results. [file 13054_2022_4116_MOESM1_ESM.docx]

**Clinical validation of a multiplex droplet digital PCR for diagnosing suspected bloodstream infections in ICU practice: a promising diagnostic tool**

Jing Wu^1†^, Bin Tang^1†^, Yuzhen Qiu^1†^, Jiang Xia^2^, Jing Zhang^2^, Ruoming Tan^1^, Jialin Liu^1^, jingjing Huang ^3^, Jieming Qu^4^, Jingyong Sun^5∗^, Xiaoli Wang^1∗^,Hongping Qu^1∗^

Online supplement

**Table S1. Detailed descriptions of the inconsistency BC-/ddPCR+ cases.**

| **variables** | | **Pathogens were identified by ddPCR*, n** | | | | **Total** |
| --- | --- | --- | --- | --- | --- | --- |
|  | **G^-^ bacteria** | | **G^+^ bacteria** | **Fungi** | 229 | |
| **Probable BSI** |  | |  |  |  | |
| Site of positive cultures |  | |  |  |  | |
| Blood culture | 15 | | 14 | 9 | 38 | |
| Nonblood culture |  | |  |  |  | |
| Abdominal | 42 | | 18 | 8 | 68 | |
| Respiratory | 15 | | 1 | 1 | 17 | |
| Skin and soft | 3 | | 0 | 0 | 3 | |
| Perianal | 7 | | 0 | 4 | 11 | |
| Urine | 0 | | 2 | 0 | 2 | |
| Multiple | 11 | | 0 | 0 | 11 | |
| **Possible BSI** |  | |  |  |  | |
| Clinical syndrome | 2 | | 3 | 1 | 6 | |
| Intra-abdominal processes | 11 | | 6 | 6 | 23 | |
| Pneumonia | 5 | | 1 | 0 | 6 | |
| Sepsis | 6 | | 5 | 1 | 12 | |
| **Putative false-positive results** | 19 | | 4 | 9 | 32 | |

* The probable and possible BSI cases were comprehensive analysed according to the microbiological data combine with clinical presentation, presumably two false-negative cases relate to *E. coli* and *C. parapsilosis* were excluded.

**Table S2. Clinical outcomes of the 150 critically ill patients according to ddPCR and BC results***

| **Diagnostic results** | **Alive, n** | **Death, n** | **Mortality, %** |
| --- | --- | --- | --- |
| ddPCR+/BC+ | 23 | 8 | 25.8 |
| ddPCR+/BC- | 31 | 11 | 26.2 |
| ddPCR-/BC- | 68 | 5 | 6.8 |
| ddPCR-/BC+ | 3 | 1 | 25.0 |

* Patients detected at least once ddpcr+ and without BC+ results were classified to the ddPCR+/BC- group.
